# Effectiveness of a PLISSIT model intervention in patients with type 2 diabetes mellitus in primary care: design of a cluster-randomised controlled trial

**DOI:** 10.1186/s12875-015-0283-0

**Published:** 2015-06-02

**Authors:** Anne Rutte, Patricia van Oppen, Giel Nijpels, Frank J. Snoek, Paul Enzlin, Peter Leusink, Petra J. M. Elders

**Affiliations:** Department of General Practice and Elderly Care Medicine, EMGO Institute for Health and Care Research, VU University Medical Centre, Amsterdam, The Netherlands; Department of Psychiatry, EMGO Institute for Health and Care Research, VU University Medical Centre, Amsterdam, The Netherlands; Department of Medical Psychology, EMGO Institute for Health and Care Research, VU University Medical Centre, Amsterdam, The Netherlands; Department of Neurosciences, Institute for Family and Sexuality Studies, KU Leuven, Leuven, Belgium; Centre for Clinical Sexology and Sex Therapy, UPC KU Leuven, Leuven, Belgium; Department of Sexology, Groene Hart Hospital, Gouda, The Netherlands; VU University Medical Centre, Department of General Practice and Elderly Care Medicine, Van der Boechorststraat 7, 1081 BT Amsterdam, The Netherlands

**Keywords:** PLISSIT, Type 2 diabetes mellitus, Sexual dysfunction, Randomised controlled trial, Primary care

## Abstract

**Background:**

Sexual dysfunction is prevalent in patients with type 2 diabetes mellitus, but remains one of the most frequently neglected complications in diabetes care. Both patients and care providers appear to have difficulty with discussing sexual problems in diabetes care. A sexual counselling model for care providers, such as the PLISSIT model, might be a useful tool to improve the discussion of sexual issues in patients with type 2 diabetes mellitus. PLISSIT stands for Permission, Limited Information, Specific Suggestions, and Intensive Therapy. Even though the use of the PLISSIT model has often been recommended in diabetes care, no evidence with regards to its effectiveness in patients with type 2 diabetes mellitus exists. This study describes the design of a cluster-randomised controlled trial evaluating the effectiveness of a PLISSIT-model intervention in men and women with type 2 diabetes mellitus in primary care.

**Methods/Design:**

Patients with type 2 diabetes mellitus, aged 40–75 years, who indicate to be dissatisfied about their sexual functioning and that they would like to talk about their sexual problem(s) with their general practitioner are recruited. All participants receive an information leaflet from the practice nurse. In the intervention group, each participant will also receive sexual counselling based on the PLISSIT model from their general practitioner. In the control group, usual care will be provided to those participants requesting an appointment with their general practitioner when the information leaflet was not deemed sufficient. Primary outcomes include sexual functioning, satisfaction about sexual function, and quality of life. Secondary outcomes are depressive symptoms, sexual distress, emotional well-being, and treatment satisfaction. Outcomes will be measured by means of self-report questionnaires at baseline, and after 3 and 12 months post-baseline. Treatment satisfaction will be assessed in telephone interviews.

**Discussion:**

This paper describes the design of a cluster-randomised controlled trial that will investigate the effectiveness of a PLISSIT-model intervention in patients with type 2 diabetes mellitus in primary care. Our study will add important and currently missing insight into the effectiveness of PLISSIT on important patient-reported outcomes of men and women with type 2 diabetes mellitus.

**Trial registration:**

Dutch Trial Registry NTR4807.

## Background

Sexual problems are highly prevalent among patients with type 2 diabetes mellitus (T2DM) [1, 2].

Although prevalence rates vary widely between studies, patients with T2DM are generally twice as often affected by sexual problems compared to persons without T2DM [3]. Men most frequently report erectile dysfunction (ED), with prevalence rates ranging from 36 to 84 % [4–9], followed by ejaculation disorders and hypoactive sexual desire disorder, 40 and 25 % respectively [7, 10]. In addition, ED is prevalent in newly diagnosed patients and is often comorbid with other sexual dysfunctions [11]. Among women, hypoactive sexual desire disorder and lubrication problems are common with prevalences of 46–82 % and 38–70 % respectively [12–15]. Women with T2DM also report arousal disorders (68 %), orgasmic disorders (38–84 %), and dyspareunia (43–46 %) [12–15].

Sexual dysfunction negatively affects the quality of life of patients with T2DM. Patients with diabetes rate their sex life as less satisfying than persons without diabetes [13, 14, 16]. Men with T2DM with ED report significantly worse emotional and psychological well-being, and more diabetes-related distress [17–19]. Even though less is known how sexual dysfunction affects the quality of life of women with T2DM, research in women with type 1 diabetes suggests a negative effect on quality of life as well [20]. Altogether, the high prevalence and negative effects of sexual dysfunction in patients with T2DM warrant clinical attention.

In the Netherlands, diabetes care is organised in primary care. Patients with T2DM generally have three-monthly control visits with the practice nurse and a yearly consultation with their general practitioner (GP). According to the Dutch Society of General Practitioners (NHG) diabetes treatment guideline, sexual issues should be addressed during this yearly appointment with the GP [21]. The GP should explicitly inquire whether the patient experiences sexual problems, including ED, loss of sexual desire, and decreased vaginal lubrication. In addition, the GP should discuss possible treatment options, such as medication adjustment [21]. Nevertheless these guidelines, sexual problems remain to be frequently neglected in diabetes care [22, 23].

Both GPs and patients experience difficulties when discussing sexual problems in clinical practice. Patients with T2DM feel embarrassed and would like the GP to initiate this discussion [23, 24], whilst GPs experience a lack of time, training, knowledge, and skills when doing so [25, 26]. Certain patient characteristics, such as gender, ethnicity, and sexual orientation, are mentioned by the GP as barriers for discussion as well [25, 26]. These difficulties impede the consultation of sexual problems in general practice. As negligence of sexual problems may only further decrease a patient’s quality of life [27], it is necessary to improve sexual counselling in diabetes care.

A sexual counselling model for care providers might be a useful tool in improving GP’s skills when discussing sexual issues with their patients. Over the past few decades, several models have been developed, such as ALARM [28], BETTER [29], and PLISSIT [30]. These models all take a somewhat different approach to sexual counselling, but have in common that little research has been conducted to study their effectiveness. The PLISSIT model, however, has shown promising features in women with sexual problems [31–33], stoma patients [34], and patients with gynaecological cancers [35, 36]. Moreover, it has been frequently recommended for health care professionals [37–39] and for diabetes care professionals specifically [3, 27, 40–42].

The PLISSIT model was developed by Annon as a framework for care providers to assist in the ordering and treatment of sexual problems [30]. PLISSIT is an acronym for the four stages of the model: Permission, Limited Information, Specific Suggestions, and Intensive Therapy. This four-step framework aides care providers with the discussion and treatment of sexual problems, whereby each step requires increasing skill and knowledge from the care professional [30]. Importantly, the use of the model is geared to the care provider’s competence, hence allowing them to refer patients for further treatment during each step.

Until now, the effectiveness of the PLISSIT model has not been examined in patients with T2DM. Considering the widespread recommendations, promising features, and beneficial findings in other patient groups, we will therefore conduct a cluster-randomised controlled trial (RCT) among patients with T2DM in Dutch primary care to examine the PLISSIT model’s effectiveness. Primary outcomes include patient-reported sexual function, satisfaction about sexual function, and quality of life. Secondary outcomes that will be considered are patient-reported depressive symptoms, emotional well-being, sexual distress, and treatment satisfaction.

## Methods

### Design and setting

This study is a cluster RCT, registered at the Dutch Trial Registry (NTR4807). The study protocol was peer-reviewed by the Dutch Diabetes Research Foundation. The study was approved by the Medical Ethics Committee of the VU University Medical Centre in Amsterdam, The Netherlands.

The study will be conducted in primary care practices in the western part of the Netherlands: the West-Friesland region, the Zaanstreek-Waterland region, and Amsterdam area. Both urban and rurally located general practices will be included. Randomisation takes place at the level of general practice. Eligible patients will either be included in the intervention or control group: structured sexual counselling based on the PLISSIT model versus unstructured sexual counselling (usual care).

### Study population

The inclusion criteria concern men and women aged 40–75 years with T2DM, who indicate to be unsatisfied about their sexual function and want to talk about their sexual problem with their GP. In addition, participants have to be able to read, write, and understand the Dutch language, which is necessary for filling out the questionnaires and talking to their GP. Participation in other scientific studies that aim to improve one or more of our outcome measures will result in study exclusion.

### Recruitment

All potential participants will be notified about the start of the study through posters in the GP’s waiting room. In addition, the practice nurse can give written information about the study. Recruitment of eligible patients is performed by the GP’s practice nurse. Given the frequent three-monthly meetings this will efficiently fit in routine care. Patients will be unaware of the practice’s randomisation status.

**Fig. 1:**
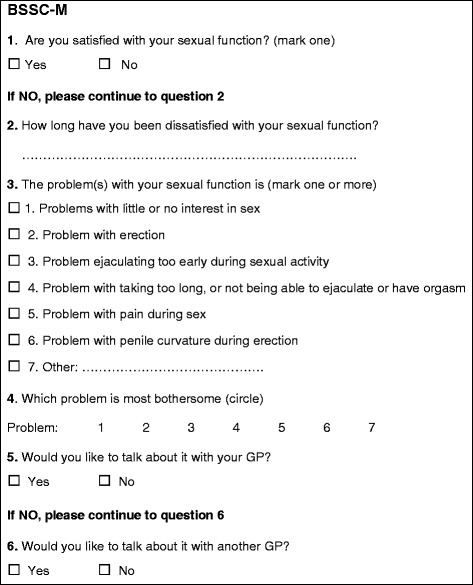
The modified Brief Sexual Symptom Checklist for Men (BSSC-M)

**Fig. 2:**
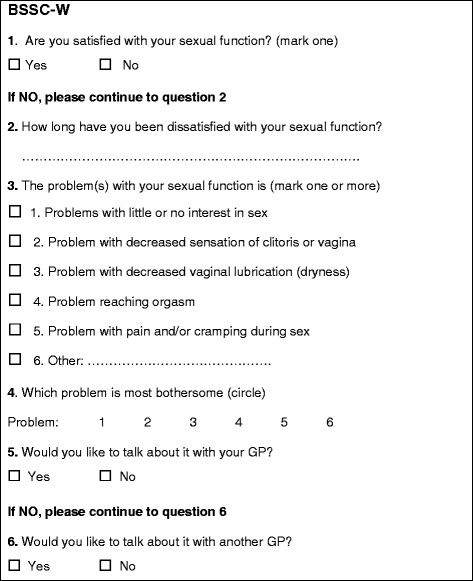
The modified Brief Sexual Symptom Checklist for Women (BSSC-W)

The practice nurse will ask all patients with T2DM aged 40–75 years to fill out a short screener questionnaire for scientific research. We use the Brief Sexual Symptom Checklist for men (BSSC-M) and women (BSSC-W) to screen for sexual dysfunction and dissatisfaction about sexual function in primary care (Fig. 1 and 2) [43]. We have slightly modified this questionnaire by changing question 5 from ‘would you like to talk about it with your doctor’ to ‘would you like to talk about it with your GP’. In addition, we added a sixth question for patients who might want to talk about their problem with another GP than their own.

Based on the results of the screener, patients are eligible for study participation if they report 1) to be sexually dissatisfied, and 2) that they would like to talk about their sexual problem with the GP. The practice nurse will explain that a scientific study program is tested in this practice, and that the particular patient is eligible for participation. Participation implies that the patient will need to fill out three questionnaires in the coming 12 months.

After handing in the baseline questionnaire, the patient receives an information leaflet from the practice nurse on Diabetes and Sexuality. In the intervention group, the practice nurse will subsequently make an appointment with the GP for each patient to talk about sexual problems. Practice nurses in the control group do not automatically make an appointment with the GP for every patient. Instead, the practice nurse will ask whether the information leaflet was of sufficient help or whether the patient would like to have an appointment with his/her GP nevertheless.

The practice nurse will fill notify the researchers if a patient consented to participate. A non-response form is filled out for those patients who do not agree to fill out the screener and/or do not want to participate in our study. The form includes the patient’s gender, year of birth, and ability to speak/read Dutch.

### Training of practice nurses

All practice nurses will receive one training session. Intervention and control practices will be trained in separate sessions to prevent contamination. The first part of the training focuses on the effects of sexual dysfunction in T2DM and about discussing sexual issues in clinical practice. Suggestions for an appropriate attitude and skills needed for introducing sexual issues are explained. This part of the training is deemed necessary for optimal recruitment of patients.

The second part of the training focuses on recruitment of eligible patients for study participation, as described above. Practice nurses will be taught to explain the study purposes, handing out the screener questionnaire, processing the results from the screener, and recruiting eligible patients for study participation.

The practice nurses will be instructed to include at least three eligible men and three eligible women per practice. The steps of the recruitment process will be practiced and references to relevant literature for further reading will be provided. The training will be given by two skilled researchers. A questionnaire to measure the practice nurse’s knowledge and competence with discussing sexual problems in patients with T2DM will be administered before and 3–4 weeks after the training.

### Intervention development

During the first step of the model, Permission, the care provider invites the patient to talk about sexual health and sexuality. One way to do so is by asking certain ‘cue questions’, such as ‘I ask all my diabetes patients about their sexuality, is it okay if I ask these questions to you now?’ or ‘People with diabetes often have questions about how diabetes may affect their sex life. Is there anything you would like to ask me?’. Permission here refers to explicit permission-giving by the patient to talk about this (yes/no) with the care provider. On the other hand, Permission also refers to the normalisation of sexual problems by the care provider (implicit permission-giving) [44]. The second step, Limited Information, concerns providing general information, for example regarding the effect of diabetes on sexual function. The first two steps of the PLISSIT model focus on the invitation to the patient to talk about sexuality and on the normalisation of the sexual problem [45].

Compared to the previous steps, the care provider needs additional skill and knowledge to carry out the third step of the model, Specific Suggestions. In order to provide relevant suggestions, the care provider needs to understand the patient’s particular complaint, which may include taking a sexual history [45]. Examples of specific suggestions may include the use of lubricants, lifestyle changes, or medication adjustment. These suggestions are aimed to directly help the patient within a relatively short period of time [45].

Since most GPs will not be skilled enough to perform the fourth step of the model, Intensive Therapy, this step will almost always include referring the patient to specialised care. Step four may be applied to complex sexual problems or problems that were not efficiently helped in the previous steps. For example, referral to a sexologist or psychologist may be necessary in case of relationship problems or sexual abuse [44]. The PLISSIT model thus provides a framework for when referral is appropriate, but most importantly, the model aids the care provider with the discrimination between problems for which brief therapy will suffice (step 1–3) and problems needing intensive therapy (step 4) [45].

### Training of GPs in the intervention group

GPs in the intervention group will receive one training session regarding the application of the PLISSIT model. General information about sexual (dys)function and T2DM will be provided. Role-play will be used to help the GPs become aware of their attitudes towards sexual health and sexuality. After the exercise, attitude and skills needed for discussing sexual problems will be discussed. The training continues with a thorough explanation of the steps and the application of the PLISSIT model, including relevant examples for each stage of the model, which may include, but are not limited to:

1. Permission: examples of certain ‘cue questions’ will be given. For instance, ‘I ask all my diabetes patients about their sexual health, it is not an easy subject to talk about, but we know that many diabetes patients suffer from sexual problems. Is it okay to talk with you about this now?’.
2. Limited Information: explanation of normal sexual functioning; explanation of the association between T2DM and sexual problems; and providing adequate additional information choosing from a variety of available information leaflets regarding sexuality and chronic illness.
3. Specific Suggestions: a training in taking a short sexual history with respect to the age, gender, ethnicity, and sexual orientation of the patient. In addition, multiple examples of ‘specific suggestions’ will be given, such as prescribing lubricants or phosphodiesterase-5 inhibitors, or providing (further) psycho-education on facilitating and impeding factors of sex.
4. Intensive Therapy: explanation of when to refer to which specialist, e.g. sexologist, psychologist, or gynaecologist. An overview of the local referral possibilities will be handed out during the training.

It will be emphasised that GPs should apply the model geared to an individual’s problems and needs, and that, consequently, not all steps are necessary for every patient. The model should not be interpreted as a linear structure, but as a dynamic process of consultation, including, when necessary, returning to previous steps or skipping one [44]. During the training, the application of the PLISSIT model will be practiced based on a theoretical case. Afterwards, references to relevant literature for further reading will be provided.

An experienced GP who also works as a certified sexologist will train the GPs in the intervention group. A questionnaire to measure the GP’s knowledge and competence with discussing sexual problems in patients with T2DM will be administered before and 3–4 weeks after the training.

### Intervention

In the intervention group, a consultation with the GP is scheduled two weeks after the meeting with the practice nurse. The GP will discuss the patient’s sexual problems according to the steps of the PLISSIT model, as described previously. Only after permission is given (step 1), the intervention will continue. In case the patient doesn’t give permission, the GP will emphasise that the patient may always raise this issue at a later moment [44]. The following steps of the model will be addressed based on the patient’s needs, allowing the GP to return to previous steps or to skip steps not deemed necessary. Since the majority of the patients will be helped by discussing the first three steps of the PLISSIT model, most of the counselling will take place in one or two consultation(s). Multiple consultations are expected to be necessary for (more) complex sexual issues in step 3 and 4.

If the GP suspects the patient to express depressive symptoms during the PLISSIT consultation, the GP will treat first what he/she deems necessary. The patient will receive care conform the NHG guideline for depressive symptoms [46]. When depressive symptoms are treated first, the GP will make a PLISSIT appointment after a clinically relevant interval.

### Control group

GPs in the control group provide care as usual [21]. At present, apart from the NHG guideline about ED [47], no NHG guideline regarding male or female sexual dysfunction exists. The GP will provide usual care conform the NHG guideline on depressive symptoms when deemed necessary [46], as previously described in the intervention strategy paragraph.

In order to establish equal referral options for both study arms, GPs in control practices will receive the same overview of specialists to whom they may refer their patients for further treatment. By providing both groups with this map we can correct for the effect a referral may have on our outcomes.

### Data collection and outcome measures

The following outcome measures are included in the questionnaires.

#### Primary outcome measures

Sexual function will be assessed with the 19-item Female Sexual Function Index (FSFI) for women [48], and the 15-item International Index of Erectile Function (IIEF) for men [49]. Both questionnaires are widely used and have been well-validated [50–52]. Satisfaction with sexual function will be measured on a 10-point Visual Analogue Scale (VAS) scale: ‘how satisfied are you with your sexual functioning on a scale from 0 to 10?’, where 0 indicates being very unsatisfied and 10 indicates being very satisfied. Quality of life will be assessed with the brief, reliable, and valid Short Form-12 item survey (SF-12) [53–55].

#### Secondary outcome measures

The 9-item Patient Health Questionnaire (PHQ-9) will be used to measure depressive symptoms [56]. The PHQ-9 has been validated in several (clinical) populations, and has shown to have good reliability, and high sensitivity and specificity for depression in patients with T2DM [57, 58]. The revised version of the Female Sexual Distress Scale (FSDS) [59], the FSDS-Revised (FSDS-R) [60], will be used to examine sexual distress. Consistent with the FSDS, the FSDS-R showed good psychometric properties for assessing sexuality-related personal distress in women [52, 59, 60]. We will use the FSDS-R to assess sexual distress in women. In addition, for exploratory purposes, we will use the FSDS-R to assess sexual distress in men. The World Health Organisation-Five Well-Being Index (WHO-5) will be used to measure emotional well-being, which has shown good psychometric properties as a screener for depression in patients with type 1 and 2 diabetes [61–63]. Finally, patients’ treatment satisfaction will be examined in qualitative interviews.

#### Process analysis

To assess whether the study protocol was followed as intended, we ask all patients at three (T1) and 12 (T2) months follow-up whether they received the information leaflet on diabetes and sexuality from the Dutch Diabetes Association and whether they had a conversation with the GP about sexual problems. In addition, we will ask about the number of return visits regarding sexual problems, and whether the patient was referred to another health care provider for this sexual problem.

A second part of the process analysis concerns qualitative research. All patients will be asked whether they may be approached for a telephone interview during the study. We will perform a purposeful sampling approach, taking the patient’s age, gender, and sexual functioning into account. An estimate of 15–20 interviews is necessary 1) to assess whether the intervention or the control procedures were performed according to the study protocol; and 2) to examine treatment satisfaction. The interviewer will be unaware of the patients randomisation status and sexual functioning.

### Sample size

We decided to settle for a power to detect an improvement of 25 % in sexual functioning, 10 % in the control group and 35 % in the intervention group. We calculated that 65 participants per group would be needed, assuming a two-sided test at an alpha of 0.05 and a power of (1 − beta) 0.90. Due to cluster randomisation at GP level, the sample size needed to be inflated based on the formula of the Design Effect: 1 + (n-1)∙ICC with n as the mean cluster size and ICC as the Intracluster Correlation Coefficient [64]. We estimate to recruit six eligible patients per practice (n=6). An ICC of 0.05 was chosen to account for the clustering of patients within practices [64, 65]. The Design Effect was calculated as 1.25. In addition, we accounted for a 20 % dropout. A total of 195 patients are needed. We will therefore aim to recruit 200 patients from 34 general practices.

### Randomisation and blinding

An independent statistician will randomly allocate the participating general practices to either one of the study arms by means of block randomisation. Practices will be matched based on location and number of patients with T2DM per practice. In addition, when necessary, we will account for practice nurses who work in multiple general practices by matching those practices in one block to ensure that practice nurses that work in different participating practices have a similar allocation in each practice. Patients will be unaware of the randomisation status, as is the analyst during the data handling and data analysis; GPs and practice nurses cannot be blinded.

### Analyses

Since we have chosen a cluster-randomised design with repeated measurements, observations and patients within a certain cluster are more alike and thus correlate to each other. We will therefore use linear and logistic multilevel regression analyses to study the effects of our trial. Data will be analysed as intention-to-treat and differences in outcomes measures between the intervention and control group will be calculated with 95 % confidence intervals. Given that not every patient in the control group will have had an appointment with their GP, a sensitivity analysis will be conducted to compare patients who had an appointment with their GP in the intervention versus control group.

## Discussion

This paper describes the design of a cluster RCT that will investigate the effectiveness of a PLISSIT model intervention in improving sexual functioning, satisfaction with sexual function, and quality of life of patients with T2DM in primary care. Secondary outcomes include depressive symptoms, emotional well-being, sexual distress, and treatment satisfaction. The results will add important and currently missing insight into the effectiveness of PLISSIT on important patient-reported outcomes of patients with T2DM.

Difficulty with discussing sexual health and sexuality in clinical practice is not limited to diabetes care, but is more wide-spread and receives little attention in research. In the literature, the necessity to address a patient’s sexual problem is frequently advocated for, for example in patients with cancer, chronic obstructive lung disease, cardiovascular disease, or multiple sclerosis [66, 67]. Although several (generic) sexual counselling models exist, these or other interventions to improve sexual counselling have been little researched thus far [31–36, 68, 69].

Compared to the other sexual counselling models [28, 29, 44], the PLISSIT model has shown beneficial effects in several patient groups [31–36]. It may therefore also function as a useful framework for GPs to discuss and treat sexual problems in diabetes care. It should, however, be emphasised that the model here is not proposed as a solution for sexual problems, but rather as a tool to improve skills and knowledge of GPs to identify and address these problems, which may result in improved patient well-being. The latter meets the requests of both GPs and patients with T2DM: improving GP’s sexual counselling skills and training on the one hand [25, 26], and integration and normalisation of care for sexual problems in diabetes care on the other hand [23].

A limitation to our study is that the external validity has to some extent been compromised by the increased internal validity. First, by specifically instructing the practice nurses in both groups to identify sexual problems, we raise more attention to the topic than usual care would normally comprise. Moreover, it is expected that GPs in the control group will also tend to pay more attention to sexual problems, although it is assumed that this effect will fade over time as the trial progresses. Second, the practice nurse implicitly performs the first step of the PLISSIT model (Permission) by handing out the screener which asks ‘Would you like to talk about it with your GP?’. This is, however, most similar to usual care where the practice nurse will screen for health problems before sending the patients to the GP.

In conclusion, the present study will evaluate the effectiveness of a PLISSIT model intervention in patients with T2DM in primary care. These findings will contribute to the field of T2DM and sexual dysfunction.

## Abbreviations

BSSC-M: Brief sexual symptom checklist for men
BSSC-W: Brief sexual symptom checklist for women
ED: Erectile dysfunction
FSDS: Female sexual distress scale
FSDS-R: Female sexual distress scale-revised
FSFI: Female sexual function index
GP: General practitioner
ICC: Intracluster correlation coefficient
IIEF: International index of erectile function
NHG: Dutch society of general practitioners
PHQ-9: 9-item patient health questionnaire
RCT: Randomised controlled trial
SF-12: Short form-12 item survey
T2DM: Type 2 diabetes mellitus
VAS: Visual analogue scale
WHO-5: World health organisation-five well-being index
